# Photodynamic Therapy with Tumor Cell Discrimination through RNA-Targeting Ability of Photosensitizer

**DOI:** 10.3390/molecules26195990

**Published:** 2021-10-02

**Authors:** Yuan Xu, Yang Tan, Xiuqin Ma, Xiaoyi Jin, Ye Tian, Miao Li

**Affiliations:** 1 College of Marine Technology and Environment, Dalian Ocean University, Dalian 116023, China; xuyuan81699@163.com; 2 Key Laboratory of Environment Controlled Aquaculture, Ministry of Education, Dalian 116023, China; 3 School of Biological Engineering, Dalian Polytechnic University, Dalian 116023, China; tanyang9808@163.com (Y.T.); maxiuqin012@163.com (X.M.); jinxiaoyi519@163.com (X.J.)

**Keywords:** photosensitizer, cyanine dyes, RNA targeting, cancer cell discrimination

## Abstract

Photodynamic therapy (PDT) represents an effective treatment to cure cancer. The targeting ability of the photosensitizer is of utmost importance. Photosensitizers that discriminate cancer cells can avoid the killing of normal cells and improve PDT efficacy. However, the design and synthesis of photosensitizers conjugated with a recognition unit of cancer cell markers is complex and may not effectively target cancer. Considering that the total RNA content in cancer cells is commonly higher than in normal cells, this study has developed the photosensitizer **QICY** with RNA-targeting abilities for the discrimination of cancer cells. **QICY** was specifically located in cancer cells rather than normal cells due to their stronger electrostatic interactions with RNA, thereby further improving the PDT effects on the cancer cells. After intravenous injection into mice bearing a xenograft tumor, **QICY** accumulated into the tumor location through the enhanced permeability and retention effect, automatically targeted cancer cells under the control of RNA, and inhibited tumor growth under 630 nm laser irradiation without obvious side effects. This intelligent photosensitizer with RNA-targeting ability not only simplifies the design and synthesis of cancer-cell-targeting photosensitizers but also paves the way for the further development of highly efficient PDTs.

## 1. Introduction

Cancer is a worldwide disease with a high mortality rate. Photodynamic therapy (PDT) is a new and emerging therapeutic method that has been recognized as a mild alternative to traditional cancer treatment methods such as chemotherapy, radiotherapy, X-ray therapy, and other combined therapies [1,2,3,4]. PDT is a typical photon-triggered therapeutic modality to cure cancer with minimal invasiveness and high spatiotemporal selectivity, which has recently attracted extensive attention from researchers [5,6,7,8,9,10]. The reactive oxygen species (ROS) released by the photosensitizer under laser irradiation are responsible for the destruction of cancer cells during PDT. The photosensitizer, which also releases ROS under laser irradiation into normal cells, is the main cause of side effects [11,12,13,14]. Hence, the design of a photosensitizer with a cancer-cell-targeting ability not only decreases side effects but improves the concentration of the photosensitizer into cancer cells, which improves the efficacy of PDT.

Cancer cell markers usually include cancer-cell-specific proteins (e.g., epithelial mucins, embryonic proteins, and glycoproteins), hormones, and enzymes [15,16,17]. The conjugation with the small molecule recognition unit of the cancer cell marker is the general method used to provide the cancer-cell-targeting ability to the photosensitizer. However, the choice of potential recognition units is complex, requiring a large amount of work concerning the structure-guided molecular design and the structure–activity relationship analysis [18,19,20]. Moreover, the design and synthesis of the conjugated photosensitizers are complicated, with many factors that should be considered. For example, the conjugation of the photosensitizer may reduce the original targeting ability of the recognition unit, in turn influencing the effects of PDT. Rather than the recognition of the cancer cell marker, RNA recognition is relatively easy to perform via electrostatic interactions with dyes [21,22]. RNA polymerase activities in cancer cells are higher than those in normal cells [23,24]. Cancer cells have higher transcription activities, shorter cell cycles, and faster growth speed [25]. Moreover, the total RNA contents in cancer cells are usually higher than those in normal cells [22]. Therefore, a photosensitizer interacting with RNA can not only specifically target tumor cells but can also generate photodynamic effects under laser irradiation, thereby realizing a highly efficient PDT with few side effects on normal cells.

Cyanine dye is widely used in multidisciplinary fields due to its absorption coefficients and fluorescence quantum yields that are high enough for fluorescence imaging and light sensing [26]. The physical properties of cyanine dyes are easily modified by acting on the cyanine structure. For example, the change in the length of the carbon chain corresponds to the bathochromic/hypsochromic shift of the absorption and emission wavelengths [27]. The adjustable properties of the cyanine dye wavelength promote the design of photosensitizers with an appropriate tissue depth for laser penetration. Furthermore, the structural modification of the heterocycles at the end of the carbon chain can alter the planar configuration of the cyanine dyes for enhancing the steric hindrance, thereby further altering the capacity for insertion between the base pairs and binding to the grooves of the DNA [21]. 

Along with these properties, and considering that cyanine dyes can both easily penetrate into cells and are highly biocompatible, their use in biological microenvironments is feasible. The positively charged cyanine dyes can interact with the negative charges in the biological microenvironments, thus forming aggregates. Cyanine dye aggregates can then accumulate into the tumor through the enhanced permeability and retention (EPR) effect [28]. Considering that RNA is a negatively charged biomacromolecule due to the existence of phosphoester bonds, once the cyanine dyes penetrate into the tumor through the EPR effects, electrostatic interactions occur between the cyanine dyes and the intracellular RNA. Furthermore, if the electrostatic interaction is the main force acting on the cyanine dyes, the cyanine photosensitizers can discriminate cancer cells that contain more RNA than normal cells under the control of the electrostatic interactions, thereby decreasing the side effects of PDT on normal cells. Since RNA stores genetic information, these cyanine photosensitizers can kill cancer cells more completely under laser irradiation, thus realizing a deep photodynamic therapy. 

In this study, the RNA-targeting ability of the cyanine photosensitizer **QICY** was developed for the discrimination and precise killing of cancer cells. **QICY** is characterized by an absorption and fluorescence emission peak in ultrapure water at 600 nm and 650 nm, respectively. In addition, the positively charged **QICY** can interact with the negatively charged RNA through electrostatic interactions. **QICY** also showed high biocompatibility in dark conditions. After the incubation of cells, **QICY** was distributed into the nucleus and mitochondria and targeted the RNA inside them. Moreover, the RNA-targeting ability of **QICY** was also proved by RNase digestion. The intracellular **QICY** fluorescence in cancer cells was much stronger than in normal cells. In addition to cancer cell discrimination, **QICY** produced a prominent photodynamic effect under 630 nm laser irradiation with a half-maximal inhibitory concentration (IC_50_) of approximately 0.20 μM. Furthermore, the in vivo photodynamic effect of **QICY** was evaluated in tumor-bearing BALB/c mice. After intravenous injection, **QICY** accumulated into the tumor through the EPR effect and, after excitation, effectively inhibited tumor growth. No abnormal change to the body weight and histology of other organs were observed after PDT treatment, excluding the side effects and systemic toxicity induced by excited **QICY**. This strategy of creating a photosensitizer with RNA-targeting ability that specifically targets cancer cells can not only decrease the complexity of the design and synthesis of the photosensitizers but can also avoid the effects of PDT on normal cells, thus providing a promising method for an effective PDT.

## 2. Results and Discussion

### 2.1. Synthesis of **QICY** and Intermediates

**Scheme 1:**
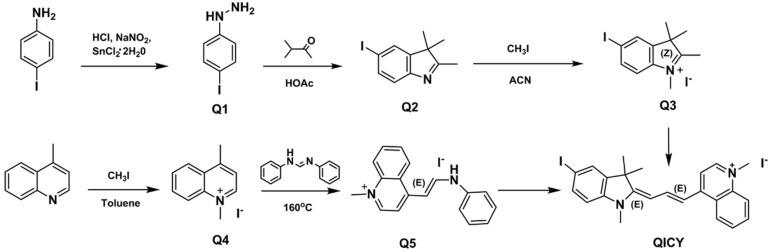
The synthetic procedure to obtain **QICY** and the intermediates *^a^*. (*^a^* The reagents and conditions are described in the Supporting Information).

Thiazole orange (TO) cyanine is always used for the detection of nucleic acids. Hence, an RNA-recognizing cyanine photosensitizer (**QICY**) was modified based on the TO skeleton. The introduction of a heavy atom on the cyanine increases the photodynamic efficacy, and the dimethyl carbon rather than the sulfur atom avoids both the insertion between DNA bases and the binding to DNA grooves [21,29,30]. These alterations made to the cyanine were made to develop a photosensitizer with RNA-recognizing ability. Firstly, a NaNO_2_ aqueous solution was added dropwise into a cooled solution containing 4-iodoaniline and hydrochloric acid, placed under continuous stirring for 30 min, and then the ice-cold SnCl_2_ solution in HCl was added. At the end of the reaction, a light brown precipitate (para-iodophenyl hydrazine, the compound Q1) was obtained. Secondly, after the refluxing of compound Q1 with acetic acid and isopropyl methyl ketone, the pH of the solution was adjusted to neutral by NaHCO_3_ to obtain the compound Q2. Then, the compound Q2 (or 4-methylquinolinium) was reacted with methyl iodide under the refluxing in toluene. The 5-iodo-1,2,3,3-tetramethyl-3H-indolium, the compound Q3 (or 1-methyl-4-methylquinolinium iodide, compound Q4), was precipitated after cooling. Then, the compound Q4 was reacted with *N*,*N*’-diphenylformamidine at 160 °C for 30 min. The resulting solid compound was further washed in ethyl ether for the purification of compound Q5, and then the compounds Q3 and Q5 were dissolved in a mixed solution of CH_2_Cl_2_ and CH_3_OH with the addition of the catalyst (Ac)_2_O and N(Et)_3_. At the end of the reaction, the precipitated **QICY** was further purified by silica column chromatography. The chemical structure, synthetic route, and compound characterization of **QICY** are shown in Scheme 1 and Figures S1–S7.

### 2.2. Spectral Characterization and RNA Interaction of QICY

**Figure 1:**
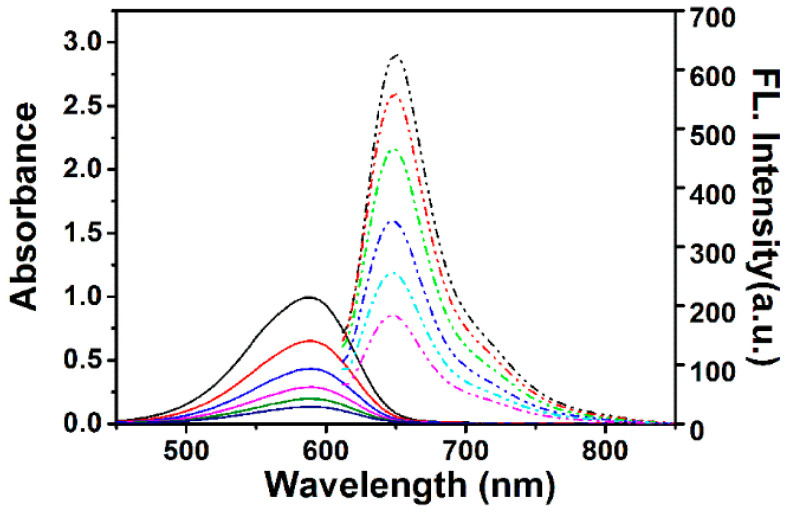
The absorption spectra and fluorescence emission spectra of **QICY** (0–20 μM) in ultrapure water (λ_ex_ = 590 nm).

**Figure 2:**
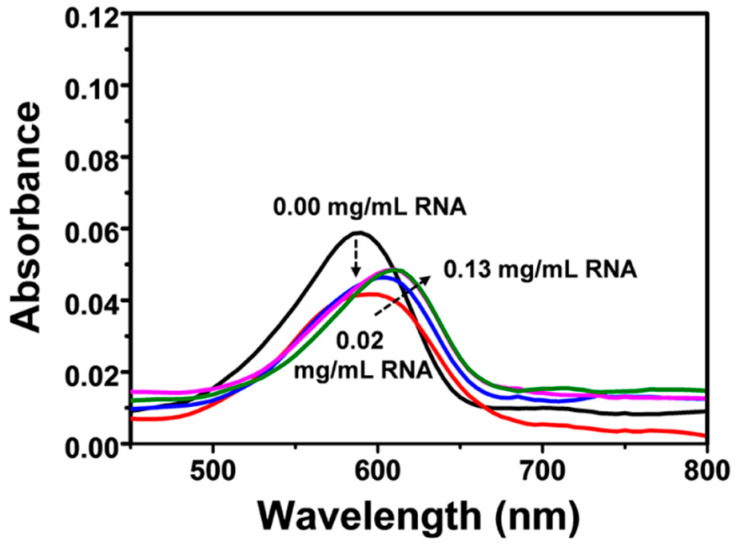
The influence of RNA titration on the absorption spectra of **QICY** (1.3 μM) in ultrapure water.

**Figure 3:**
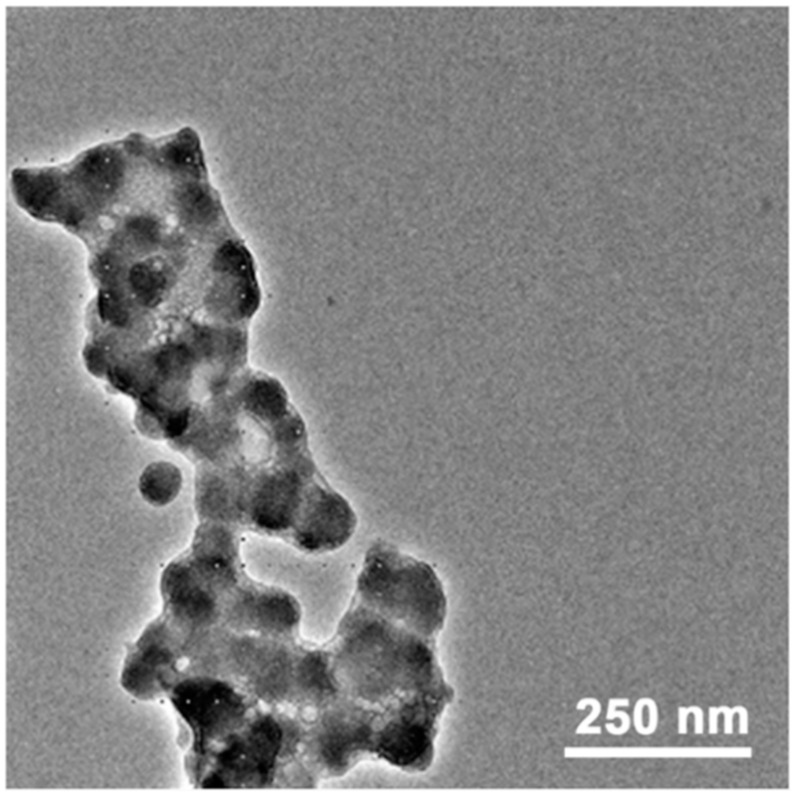
The morphology of **QICY** after interaction with RNA in ultrapure water as visualized by TEM.

**Figure 4:**
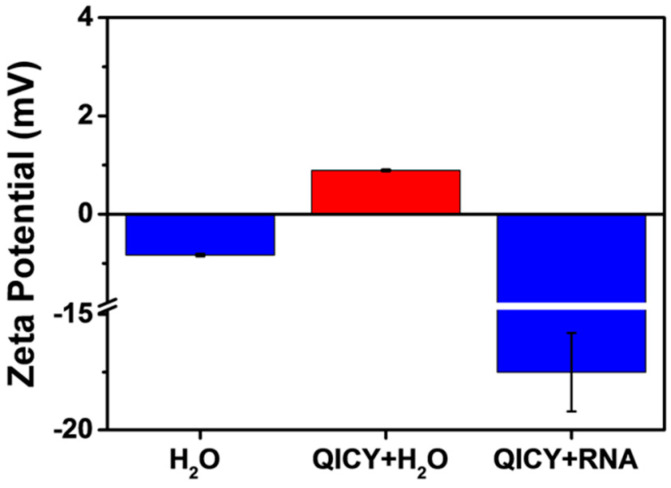
The zeta potentials of ultrapure water, **QICY** in ultrapure water, and **QICY** after interaction with RNA in ultrapure water.

UV-Vis absorption and the fluorescence spectra of **QICY** were measured in ultrapure water to determine the location of the peaks of absorption and fluorescence emission (Figure 1). The absorption peak of **QICY** was located at 600 nm. The absorbance of **QICY** increased with the increasing concentration in ultrapure water (Figure 1). Meanwhile, the intensity of the fluorescence emission peak at 650 nm was also enhanced with the increasing concentration of **QICY** (Figure 1). However, the absorbance of **QICY** in ultrapure water first decreased as the RNA was added and then increased with the continuous addition of the RNA (Figure 2 and Figure S8). The addition of the RNA initially attenuated the absorbance of **QICY**, then increased the **QICY** absorbance, indicating that there were interactions between **QICY** and RNA in ultrapure water. A transmission electron microscope (TEM) showed that **QICY** (1.3 μM) formed nanoparticles in ultrapure water under the control of RNA (0.02 g/mL) (Figure 3 and Figure S9). Hence, the aggregation of **QICY** with RNA induced the initial decrease of the absorbance, while the excess of RNA interacting with less **QICY** induced the disaggregation of **QICY** to a certain extent, which in turn increased the absorbance of **QICY**. In Figure 4, the zeta potentials of **QICY** (+0.89 mV) and **QICY** + **RNA** (−17.5 mV) in ultrapure water revealed the electrostatic interactions between the positively charged **QICY** and the negatively charged RNA. The electrostatic interactions between **QICY** and RNA provided the possibility for specifically targeting the RNA in cells.

### 2.3. Subcellular Localization of QICY

**Figure 5:**
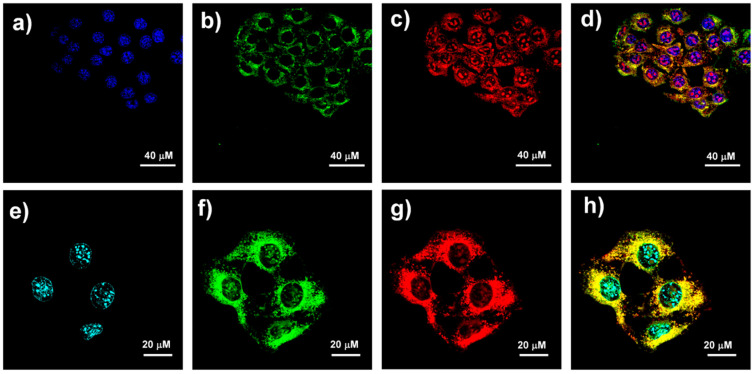
The colocalization assay by the CLSM. 4T1 cells stained with: (**a**) Hoechst 3342; (**b**) Mito-tracker Green; (**c**) **QICY**, which were separately imaged to acquire the merged picture (**d**). 4T1 cells stained with: (**e**) Hoechst 3342; (**f**) SYTO^®^ RNASelect™; (**g**) **QICY**, which were separately imaged to acquire the merged picture (**h**).

**Figure 6:**
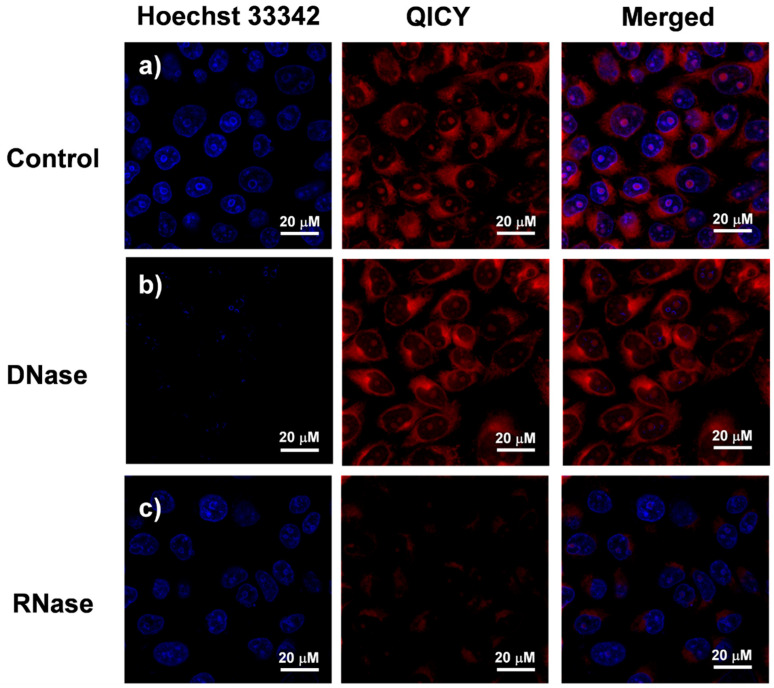
The fluorescence intensity of Hoechst 3342 and **QICY** in fixed MCF cells treated with RNase or DNase: (**a**) Control cell group; (**b**) DNase treatment group; (**c**) RNase treatment group stained with Hoechst 33,342 and **QICY**.

The RNA-targeting ability of **QICY** was monitored by performing colocalization experiments using commercial positioning dyes and confocal laser scanning microscopy (CLSM). The blue-emitting fluorescent compound Hoechst 33,342 is specific for nuclear DNA, even in the presence of RNA, while Mito-tracker^®^ Green FM is widely used to label mitochondria in living cells. The localization of **QICY** in the cell organelles was evaluated by the simultaneous culture of 4T1 cells with **QICY**, Hoechst 33,342, and Mito-tracker^®^ Green FM. The CLSM results in 4T1 cells demonstrated that **QICY** was localized in the mitochondria and nucleus (Figure 5a–d), thereby revealing the mitochondrial and nuclear targeting ability of **QICY**. The fluorescence imaging of **QICY**, Hoechst 33,342, and SYTO^®^ RNASelect™ (a cell-permeant nucleic acid stain that selectively stains RNA) in Figure 5e–h showed that **QICY** was localized into the mitochondrial and nucleoli RNA in 4T1 cells. The RNA-targeting ability of **QICY** was further supported by RNase and DNase treatments in MCF-7 cells (Figure 6). The fluorescence of **QICY** in the control group without RNase and DNase treatment was distributed in the nucleoli and mitochondria. However, compared to the control group, no significant change of **QICY** fluorescence intensity was found in the DNase treatment group, while the fluorescence of Hoechst 3342 in the cell nucleus disappeared. In contrast, **QICY** fluorescence intensity in the nucleoli and mitochondria in the RNase treatment group decreased compared to the control cells. Thus, **QICY** offered the possibility to enter cells that contained more RNA due to its RNA-targeting ability.

### 2.4. Cancer Cell Discrimination Assay

**Figure 7:**
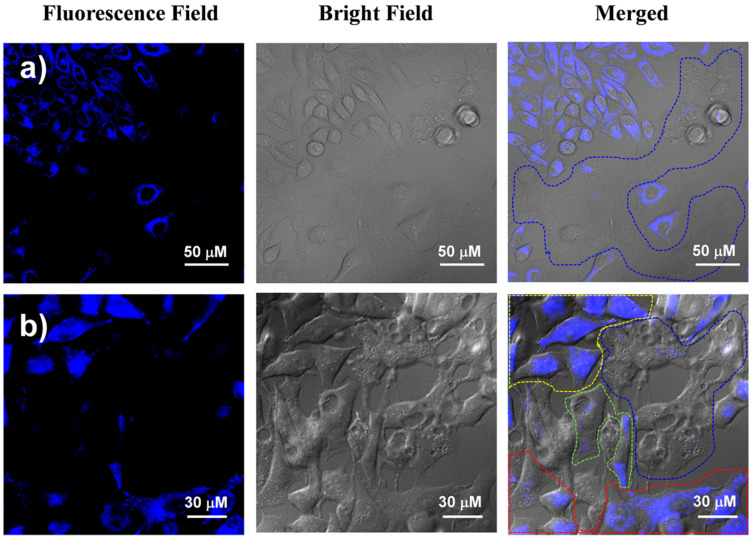
The CLSM images of (**a**) MCF-7 and COS-7 cells; (**b**) MCF-7, 4T1, and COS-7 cells cocultured with **QICY**.

In order to accurately assess the cancer-cell-targeting ability of **QICY**, cancer cells and normal cells were cocultured in an adjacent but different position in the same dish to create the environment of the tumor surrounded by normal tissues. Distinguishable morphology differences among the cell types, the same cell densities, and an identical culture environment are all necessary prerequisites to perform this specific coculture experiment. Hence, MCF-7 cells were cocultured with COS-7 cells, and MCF-7 and 4T1 were cocultured with COS-7 cells, while the selective recognition of **QICY** towards cancer cells was tested. In the coculture environment composed of MCF-7 and COS-7 cells, the COS-7 cells were located among the MCF-7 cell population **(**Figure 7a). However, **QICY** fluorescence intensity in COS-7 cells of irregular shapes (the blue dash line area) was not as large as the surrounding spindle MCF-7 cells **(**Figure 7a). The fluorescence intensity of **QICY** in MCF-7 cells was greater than in COS-7 cells, indicating that cancer cells could be successfully targeted by **QICY**. Moreover, a coculture experiment among the three types of cells was performed to further confirm the cancer-cell-targeting ability of **QICY** (Figure 7b) via the coculture of MCF-7 and 4T1 cells (cancer cells) with COS-7 cells (normal cells) in the same dish. Stereoscopic and spindle-like MCF-7 cells, polygonal and thin slice-like 4T1 cells, and irregularly shaped COS-7 cells were surrounded by yellow, red, and blue dash lines, respectively, in Figure 7b. The fluorescence intensities inside the yellow and red lines (average fluorescence intensity: 697 and 531) were different from those inside the blue dash line, suggesting that **QICY** distinguished cancer cells from normal cells due to its selective entry into the cancer cells. In addition to the above-mentioned three regions of the cells, the fluorescence intensity in isolated MCF-7 cells grown together with COS-7 cells (the green dash line; average fluorescence intensity: 128) was much weaker than in the groups of MCF-7 cells (the yellow dash line). Therefore, the average fluorescence intensity of **QICY** in cancer cells was positively associated with the interaction of cancer cells with **QICY** per unit volume. The stronger the interactions, the more photosensitizers selectively entered the tumor cells. The RNA transcription activity and the total RNA content in cancer cells are relatively higher than those in normal cells (such as COS-7) due to their fast growth speed [22]. Thus, cancer cells with more of the RNA than the normal cells, together with the ability of **QICY** to selectively target RNA, could be the potential reason for the ability of **QICY** to discriminate between cancer cells and normal cells.

### 2.5. In Vivo Tumor Imaging and Therapy

**Figure 8:**
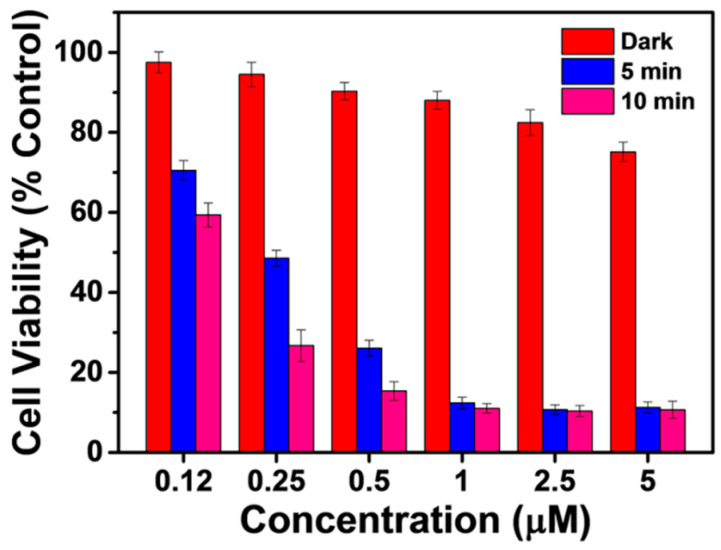
The cell viability of 4T1 cells incubated with **QICY** at different concentrations and irradiated by 630 nm laser (Laser power density: 20 mW/cm^2^; Irradiation time: 5 or 10 min).

**Figure 9:**
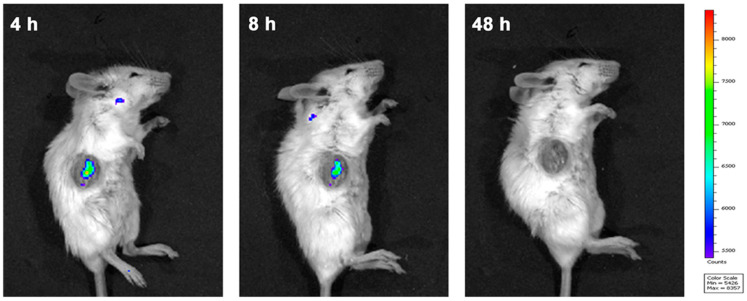
In vivo fluorescence imaging of 4T1 tumor-bearing BALB/c mice after the intravenous injection of **QICY** at the concentration of 400 μM, Ex. = 630 nm/Em. = 700 nm.

**Figure 10:**
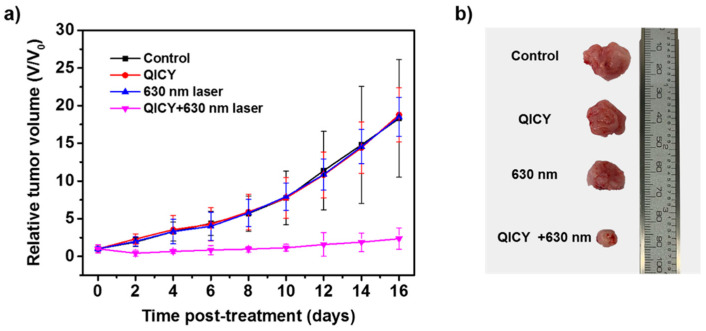
Comparison of the relative tumor volume after treatments: (**a**) Relative tumor volume of the mice in the control, **QICY**, 630 nm laser irradiation, and **QICY** + 630 nm laser irradiation groups after the different treatments (*n* = 5 per group); (**b**) Representative tumor images in the different treatment groups on day 16 after treatment.

**Figure 11:**
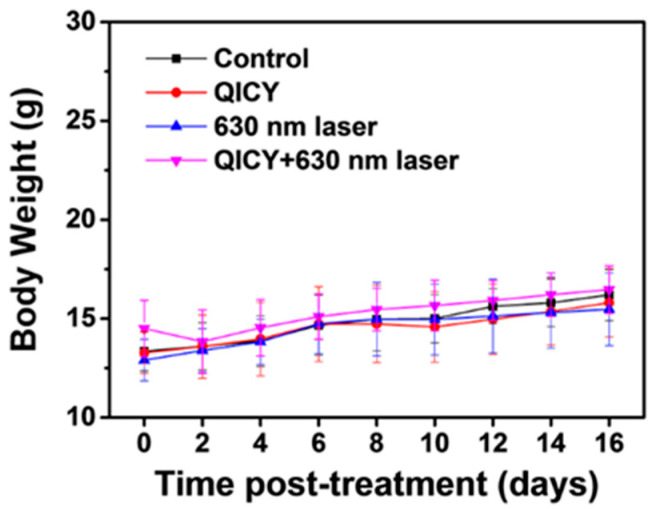
The body weights of the mice in the control, **QICY**, 630 nm laser irradiation, and **QICY**+ 630 nm laser irradiation groups after the different treatments (*n* = 5 per group).

**Figure 12:**
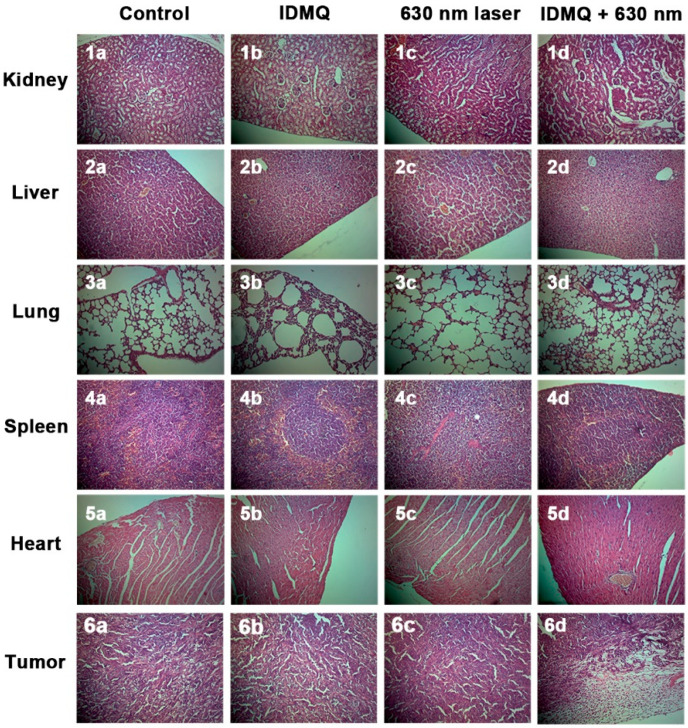
Images of H&E staining of the main organs in the control, 630 nm laser irradiation, **QICY**, and **QICY** + 630 nm groups on day 16 after treatment.

The cancer cell discrimination ability of **QICY** may allow for effective anticancer treatment by avoiding the destruction of normal cells. **QICY** also had a high photostability (Figure S10). The photodynamic treatment effect of **QICY** was evaluated by (3-(4,5-dimethylthiazol-2-yl)-2,5-diphenyltetrazolium bromide) (MTT) assays. **QICY** significantly killed cancer cells under 630 nm laser irradiation (Figure 8). In contrast, the dark toxicity of **QICY** was scarce, thereby demonstrating that cancer cell death was mainly due to the photodynamic effects of **QICY**. In addition, the IC_50_ values (20 mW/cm^2^, 10 min: 0.24 μM; 20 mW/cm^2^, 5 min: 0.19 μM) proved the effectiveness of PDT using **QICY**. The in vivo photodynamic effect of **QICY** was also evaluated in female BALB/c mice carrying 4T1 subcutaneous tumor xenografts. **QICY** was injected into the mice via the tail vein after anesthesia. Then, **QICY** in the blood stream was monitored by an in vivo fluorescence imaging system (Ex. = 630 nm/Em. = 700 nm). The peak fluorescence intensity appeared at 4 h, and **QICY** accumulation into the tumor ended at 48 h (Figure 9, Figures S11 and S12). The tumor-targeting time of **QICY** could be used for the determination of the optimal time of administration in a subsequent PDT treatment, thereby maximizing the concentration of **QICY** under laser irradiation. Then, BALB/c mice subcutaneously carrying a 4T1 tumor were randomly divided into control, 630 nm laser irradiation, **QICY**, and **QICY** + 630 nm groups (*n* = 5 per group) in order to objectively evaluate the PDT effect of **QICY**. The doses of **QICY** and the laser intensity in the above groups were 1.73 mg/kg and 80 mW/cm^2^, respectively. The tumor volume in the control, 630 nm laser irradiation, and **QICY** groups were evaluated at the end of the treatment, revealing that these three groups had nearly 18 times the original tumor volume (Figure 10). In contrast, the tumor volume in the **QICY** + 630 nm PDT group was less than that in the control and other experimental groups. This result suggested that **QICY** could effectively inhibit the growth of the tumor under 630 nm laser irradiation (Figure 10). Moreover, no abnormal changes in body weight were observed after PDT (Figure 11). Hematoxylin and eosin (H&E) staining was performed to evaluate the conditions of the main organs. A histological difference was observed between PDT and the control group, proving that **QICY** under 630 nm effectively killed cancer cells (Figure 12). However, no cell necrosis or inflammation lesions were observed in the kidneys, livers, lungs, spleens, or hearts of the mice in the PDT and other experimental groups (Figure 12). Therefore, this in vivo experiment suggested that **QICY** could effectively treat tumors under 630 nm laser irradiation, simultaneously excluding the side effects and the systemic toxicity of **QICY** + 630 PDT.

## 3. Materials and Methods

### 3.1. Materials

All the reagents used in this study were purchased from Shanghai Sahn Chemical Technology Co., Ltd. (Shanghai, China) unless otherwise stated. The reagents were not further purified before use. However, the solvents were purified in accordance with standard methods, and the purified water was twice-distilled by a Milli-Q system. Flash column chromatography (200–300 mesh silica gel, Qingdao Ocean Chemicals) was performed for the purification of the synthetized intermediates and **QICY**. Nuclear magnetic spectra of synthetized compounds were recorded by a Bruker spectrometer using TMS as the internal standard. In addition, mass spectrometry of **QICY** was acquired using an LC/Q-TOF-MS instrument (Palo Alto, CA, USA). The colocalization assay of **QICY** in cells was performed using Hoechst 3342, Mito-tracker Green, and SYTO^®^ RNASelect™ purchased from KeyGen Biotech (Nanjing, China).

### 3.2. Detection of Spectroscopic Properties

Fluorescence and UV-Vis absorption spectra were detected using Agilent Technologies fluorescence and a UV-Vis spectrophotometer (Palo Alto, CA, USA), respectively. Excitation and emission slit widths of the fluorescence spectrophotometer were adjusted for acquiring the suitable fluorescence intensity. The slit widths of different experimental groups were kept to a constant value in the same assay. The concentration of **QICY** obeyed the Beer–Lambert Law to clearly evaluate the spectral change. Furthermore, the absorption spectra of **QICY** in the titration assay of the RNA were recorded by using RNA at different concentrations dissolved in PBS.

### 3.3. Cell Culture and Simulation of Tumor Environment

Human breast cancer cells (MCF-7 cells), cercopithecus aethiops kidney cells (COS-7 cells), and murine mammary carcinoma cancer cells (4T1 cells) were purchased from KeyGen Biotech (Nanjing, China). MCF-7, COS-7, and 4T1 cells were cultured in Dulbecco’s modified Eagle’s medium (DMEM, Gibco), supplemented with 10% fetal bovine serum (PAN) and 1% penicillin/streptomycin (Hyclone) and incubated at 37 °C under 5% CO_2_ and 95% air. After cell digestion, cells were seeded in a confocal glass-bottom dish at a density of 10^5^ cells per dish. The different types of cells were seeded in adjacent but different areas of the cell dish so to simulate the tumor surrounded by normal tissues. Cells were allowed to adhere to the bottom of the dish for 24 h in a complete culture medium before confocal imaging.

### 3.4. Establishment of the Animal Model

The proliferation and metastasis of 4T1 tumor cells in BALB/c mice is similar to human breast cancer. Hence, 4T1 tumors in BALB/c mice are usually used as an animal model for the simulation of this type of cancer. Female BALB/c mice (4–6 weeks old, weighing approximately 12 g) were purchased from a Specific Pathogen Free (SPF) Experimental Animal Center of Dalian Medical University (Dalian, China) and were used for the establishment of a subcutaneous 4T1 tumor model. The animal protocols used in this study were approved by the local research ethics review board of the Animal Ethics Committee of Dalian University of Technology (Ethics approval no. 20-043). The 4T1 tumor implants into the mice were obtained by the inoculation of 4T1 cells on the right flank of each mouse, at a density of approximately 1×10^7^ cells in 100 μL PBS per mouse. The tumor was monitored 2 days after the injection of the tumor cells, and the tumor volume (*V*) was calculated by the formula *V* = *L*/2 × *W* ^2^ after measuring the length (*L*) and width (*W*) by a caliper [31].

## 4. Conclusions

The photosensitizer **QICY** with an RNA-targeting ability was created with the aim of discriminating cancer cells from normal cells and thus performing a precise and efficient photodynamic treatment under laser irradiation. The absorbance and fluorescence emission peaks of **QICY** were localized at 600 nm and 650 nm, respectively. **QICY** interacted with RNA via electrostatic interactions and targeted mitochondrial and nuclear RNA in the cells. Since the content of RNA in cancer cells is more than in normal cells, **QICY** distinguished cancer cells even when they were cocultured with normal cells. Based on this property, **QICY** generated an evident PDT effect under 630 nm laser irradiation and can avoid the killing of normal cells. After intravenous injection, **QICY** accumulated in the tumor through EPR effects, automatically targeted cancer cells under the control of RNA, and effectively inhibited tumor growth under 630 nm laser irradiation. The H&E staining of the tumor further demonstrated the PDT efficacy of **QICY**, while the side effects and systemic toxicity of **QICY** during the PDT were not observed. Therefore, this photosensitizer, which automatically discriminates cancer cells from normal cells through electrostatic interactions with RNA, not only simplifies the design and synthesis of the photosensitizers but also improves its PDT efficacy, thereby offering a promising approach for a precise and highly efficient PDT in clinical practice.

## Data Availability

Not applicable.

## Supplementary Materials

The following are available online. Table S1: Imaging conditions of QICY, RNA stain, and Hoechst 3342 in CLSM. Figure S1: 1H-NMR spectrum of compound Q1 recorded in CDCl3; Figure S2: 1H-NMR spectrum of compound Q3 recorded in DMSO-d6; Figure S3: 1H-NMR spectrum of compound Q4 recorded in DMSO-d6; Figure S4: 1H-NMR spectrum of compound Q5 recorded in DMSO-d6; Figure S5: 1H-NMR spectrum of **QICY** recorded in DMSO-d6; Figure S6: 13C-NMR spectrum of **QICY** recorded in DMSO-d6; Figure S7: ESI mass spectrum of compound **QICY**; Figure S8: Influence of RNA titration on the fluorescence emission spectra of **QICY** (1.3 μM) in ultrapure water; Figure S9: No **QICY** aggregates observed by TEM before interaction with RNA in ultrapure water; Figure S10: Absorbance change of **QICY** and DPBF in DMSO with irradiation time; Figure S11: In vivo fluorescence imaging of 4T1 tumor-bearing BALB/c mice after the intravenous injection of **QICY** at the concentration of 400 μM. Ex. = 630 nm/Em. = 700 nm; Figure S12: Fluorescence imaging of various organs (from top to bottom, left to right: tumor, kidney, colon, lung, heart, spleen, and liver) at 4 h after the intravenous injection of **QICY** (tail vein injection, 400 μM, 100 μL, 1.73 mg/kg).
