# Supplementary material for: Photodynamic Therapy with Tumor Cell Discrimination through RNA-Targeting Ability of Photosensitizer
Source: Molecules. 2021 Oct 2;26(19):5990. doi: 10.3390/molecules26195990 (PMC8512109; doi:10.3390/molecules26195990)
Supplement: Supplementary file 1 [file molecules-26-05990-s001.zip › molecules-1382452-supplementary.pdf]

Supporting Information

Tumor cell-discrimination photodynamic therapy through RNA-targeting ability of photosensitizer

Yang Tan [2,†], Yuan Xu [1,3,†], Xiuqin Ma[2], Xiaoyi Jin [2], Miao Li[2,*], Ye Tian[1,3*]

[1] College of Marine Technology and Environment, Dalian Ocean University, Dalian 116023, China; xuyuan81699@163.com (Y. X.)

[2] School of Biological Engineering, Dalian Polytechnic University, Dalian 116023, China; tanyang9808@163.com (Y. T.); maxiuqin012@163.com (M. X.); jinxiaoyi519@163.com (J. X.)

[3] Key Laboratory of Environment Controlled Aquaculture, Ministry of Education, Dalian 116023, China; xuyuan81699@163.com

* Correspondence: tianye@dlou.edu.cn (Y.T.); lim@mail.dlut.edu.cn (M.L.);Tel.: +86-0411-8476-3255 (Y.T.); +86-0411-8632-2228 (M.L.)

[†] These authors contributed equally to this work.

1. Synthesis of QICY and Intermediates

*1.1 Synthesis of Compound **Q1***

This compound Q1 was prepared according to the published procedure.[1] The solution of 4-iodoaniline (10.95 g, 49.99 mmol) and hydrochloric acid solution was cooled to -10°C and then $NaNO_2$ aqueous solutions (3.45 g, 49.99 mmol, 15 mL) was added drop wise with continuous stirring. At -10°C, the reaction mixture was stirred for 0.5 h and remained stationary for further 0.5 h. After reaction, the precipitate was washed with water and poured into 100 mL H_2O. Then, the solution was adjusted to pH=13 with saturated NaOH solution. The product filtered from the solution was further purified through a column chromatography using CH_2Cl_2 to collect the brown needles, **Q1** (6.50 g, 55.55%).

^{1}H NMR (400 MHz, $CDCl_3$) δ 7.49 (d, J = 8.8 Hz, 2H), 6.62 (d, J = 8.8 Hz, 2H), 5.18 (s, 1H), 3.55 (s, 2H).

HRMS-ESI: m/z cald $[M+H]^+$ for $C_6H_8IN_2^+$, 234.9724; found, 234.9727.

*1.2 Synthesis of Compound **Q3***

This compound **Q3** was prepared according to the published procedure.[2] The mixture of *iso*-propylmethyl ketone (0.66 g, 7.63 mmol) and *para*-iodophenyl hydrazine (1.05 g, 4.49 mmol) was refluxing under the catalysis of acetic acid (10 mL) in the N_2 atmosphere for 5 h. After reaction, the

solution diluted with H_2O was neutral to pH=7 by $NaHCO_3$. Then, the indole **Q2** was extracted with ethyl acetate, yielding a brown oil (1.10 g, 3.86 mmol, 86% crude). And then **Q2** reacted with iodomethane (0.87 g, 6.10 mmol) in acetonitrile (10 mL) under refluxing at 70°C overnight. The resulting precipitate was filtered, washed with diethyl ether to yield **Q3** as a pink solid (0.29 g, 67%).

^{1}H NMR (500 MHz, DMSO-d_6) δ 8.29 (s, 1H), 8.01 (d, J = 8.4 Hz, 1H), 7.72 (d, J = 8.4 Hz, 1H), 3.93 (s, 3H), 2.73 (s, 3H), 1.52 (s, 6H).

HRMS-ESI: m/z cald $[M]^+$ for $C_{12}H_{15}IN^+$, 300.0244; found, 300.0242.

1.3 Synthesis of Compound ***Q4***

Mixing lepidine (2.00 g, 13.97 mmol) with methyl iodide (9.91 g, 69.84 mmol) in 20 mL toluene under refluxing for 8 h can obtain 1-methyl-4-methylquinolinium iodide **Q4**.[3] After cooling the solution, the solid product was firstly crushed, then washed several times with ethyl ether, and then dried under vacuum to give yellow product **Q4**.

^{1}H NMR (500 MHz, DMSO-d_6) δ 9.36 (d, *J* = 6.0 Hz, 1H), 8.54 (d, *J* = 9.1 Hz, 1H), 8.49 (d, *J* = 8.9 Hz, 1H), 8.28 (t, *J* = 7.9 Hz, 1H), 8.07 (dd, *J* = 13.0, 6.7 Hz, 2H), 4.58 (s, 3H), 3.01 (s, 3H).

HRMS-EI: m/z cald $[M]^+$ for $C_{11}H_{12}N^+$, 158.0964; found, 158.0964.

1.4 Synthesis of Compound ***Q5***

Compound **Q4** (1.80 g, 6.32 mmol) reacted with N,

N′-diphenylformamidine (1.24 g, 6.32 mmol) at 160°C for 30 min.[3] Then, the resulting precipitate was washed several times with ethyl ether. And then the dry powder was further crystallized from ethanol to obtain **Q5** in fair purity (1.79 g, 4.61 mmol, 73% yield).

^{1}H NMR (500 MHz, DMSO-*d*$_6$) δ 10.94 (d, *J* = 11.9 Hz, 1H), 8.89 (t, *J* = 12.2 Hz, 1H), 8.66 (d, *J* = 7.0 Hz, 1H), 8.32 (d, *J* = 8.5 Hz, 1H), 8.14 (dd, *J* = 7.7, 4.9 Hz, 2H), 8.10 – 8.05 (m, 1H), 7.84 (t, *J* = 7.6 Hz, 1H), 7.48 – 7.39 (m, 4H), 7.13 (t, *J* = 7.1 Hz, 1H), 6.75 (d, *J* = 12.4 Hz, 1H), 4.24 (s, 3H).

HRMS-ESI: m/z cald [M]$^+$ for $C_{18}H_{17}N_2^+$, 261.1386; found, 261.1384.

1.5 Synthesis of Compound ***QICY***

Firstly, **Q3** (426.84 mg, 1.00 mmol) and **Q 5** (388.05 mg, 1.00 mmol) were dissolved in the mixed solutions of CH_2Cl_2/CH_3OH (V_1/V_2=1:1). Then, 1 mL of $(Ac)_2O$ and 1 mL $N(Et)_3$ were added as the catalyst in to the solutions with stirring at room temperature for 1.5 h. After reaction, the precipitated dye **QICY** was washed by high volumes of ethyl ether. And the crude dye **QICY** was further purified by silica column chromatography using the mixture of CH_2Cl_2/CH_3OH (V_1/V_2=200:1200/) mixture as eluent. Meanwhile, the blue fraction was collected, yielding green solid (209.50 mg, 0.35 mmol, 35% yield).

^{1}H NMR (500 MHz, DMSO-*d*$_6$) δ 8.65 (dd, *J* = 15.5, 7.7 Hz, 2H), 8.29 (t, *J* = 13.2 Hz, 1H), 8.18 (dd, *J* = 10.7, 8.0 Hz, 2H), 8.09 (t, *J* = 7.4 Hz, 1H), 7.88 – 7.82 (m, 2H), 7.63 (d, *J* = 9.8 Hz, 1H), 7.34 (d, *J* = 13.8 Hz, 1H), 7.00 (d, *J* = 8.3 Hz, 1H), 6.17 (d, *J* = 12.5 Hz, 1H), 4.30 (s, 3H), 3.32 (s, 3H), 1.68 (s, 6H).

^{13}C NMR (101 MHz, DMSO-d_6) δ 166.65, 151.77, 144.76, 143.36, 142.49, 142.12, 138.79, 136.48, 133.85, 130.61, 127.63, 125.21, 124.65, 118.59, 113.28, 111.85, 111.26, 100.03, 85.41, 47.17, 45.73, 42.99, 29.88, 27.94.

HRMS-ESI: m/z cald $[M]^+$ for $C_{24}H_{24}IN_2^+$, 467.0979; found, 467.0974.

2. Structure Analyzing and Confirmation ^1H-NMR, ^{13}C-NMR, and TOF-MS Spectra of QICY and Intermediates

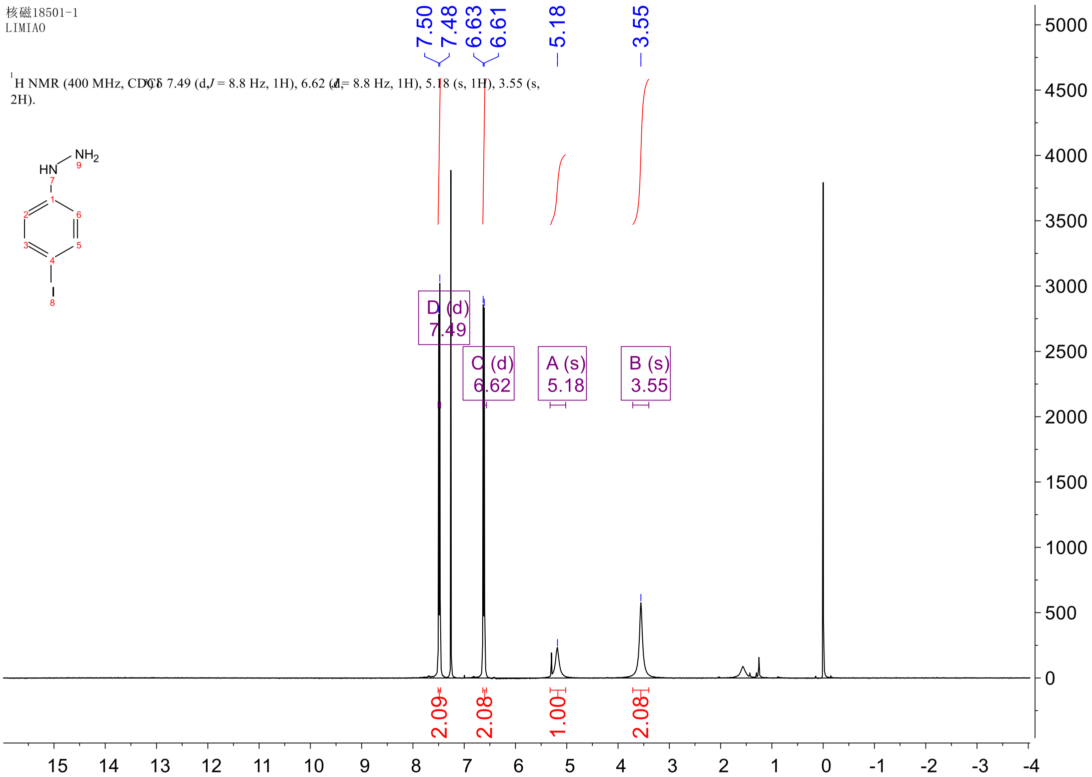

Figure S1. ^1H NMR spectrum of compound **Q1** recorded in $CDCl_3$.

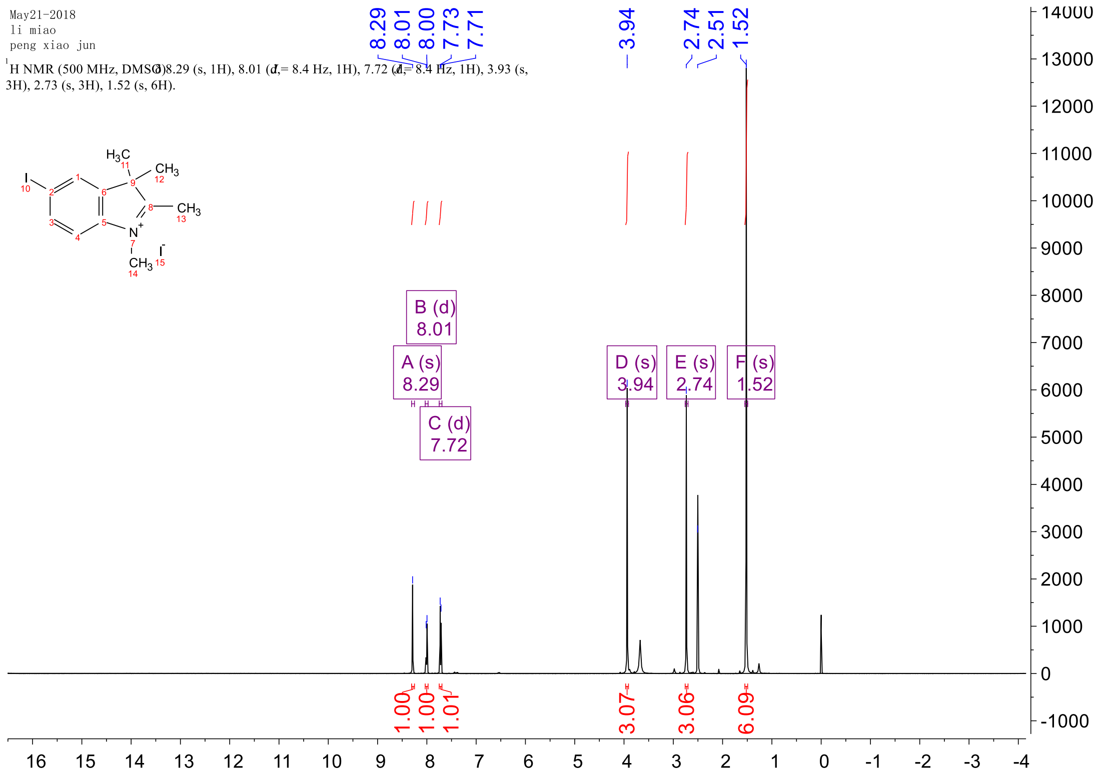

Figure S2. ^{1}H NMR spectrum of compound **Q3** recorded in DMSO-*d*$_6$.

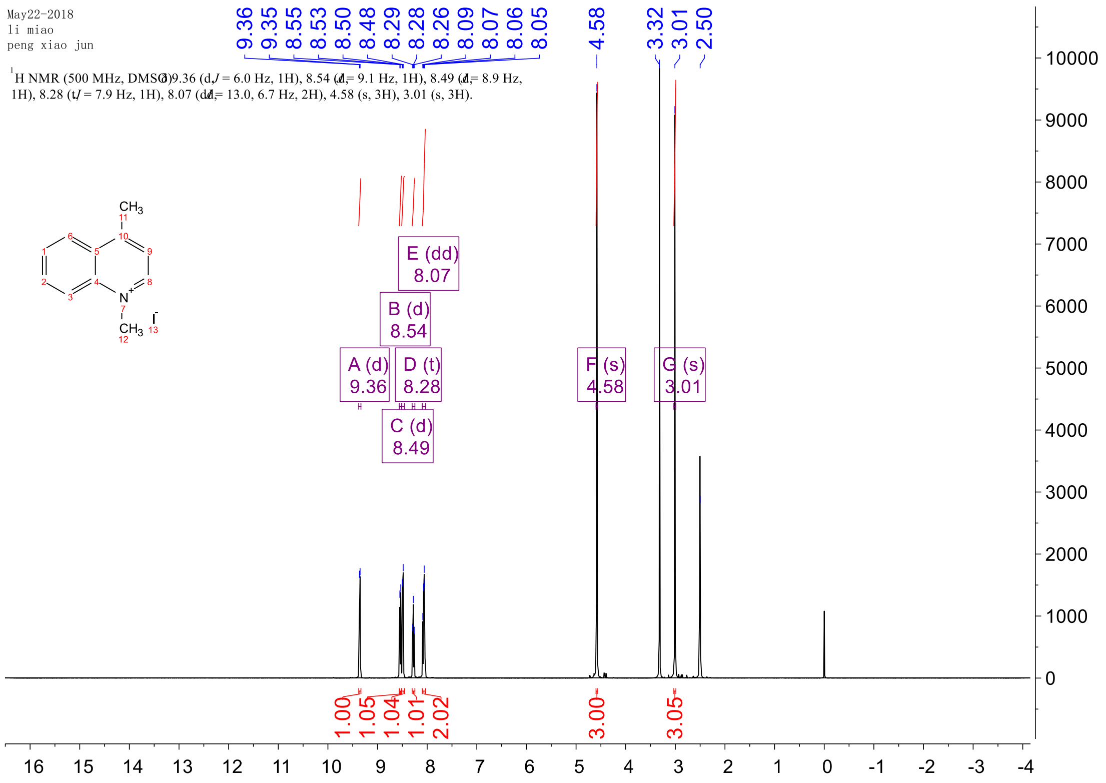

Figure S3. ^{1}H NMR spectrum of compound **Q4** recorded in DMSO-*d*$_6$.

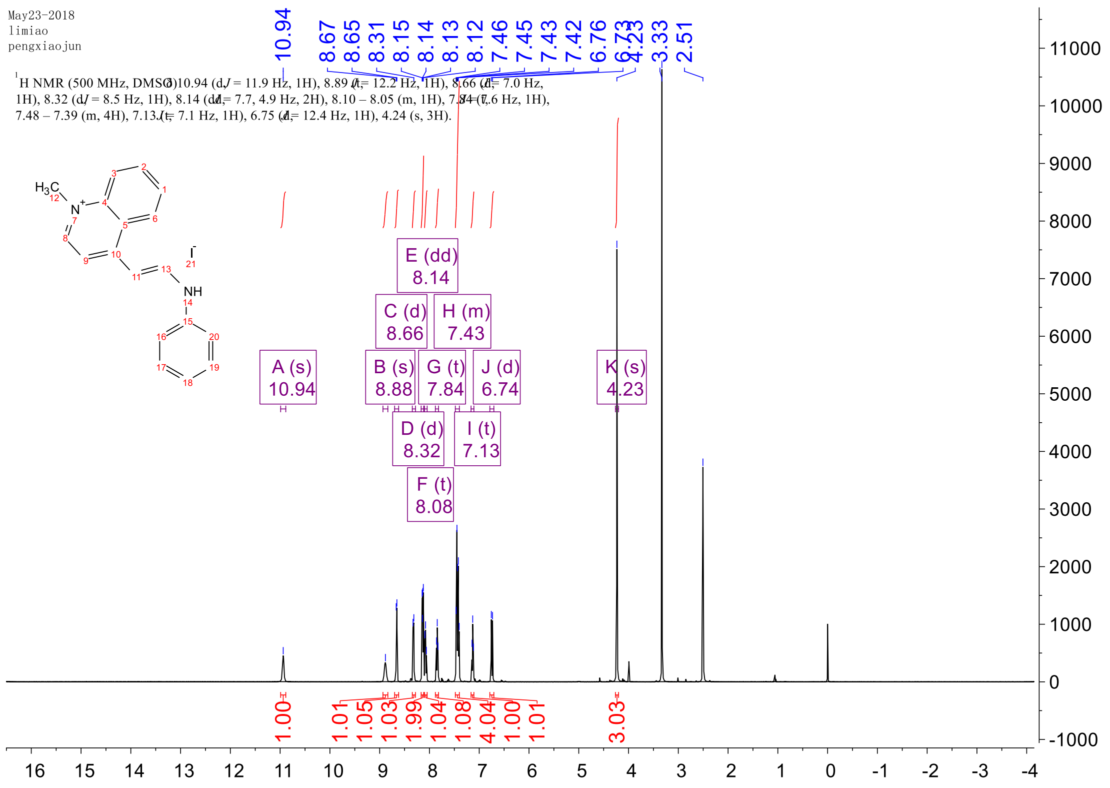

Figure S4. ¹H NMR spectrum of compound **Q5** recorded in DMSO-*d*₆.

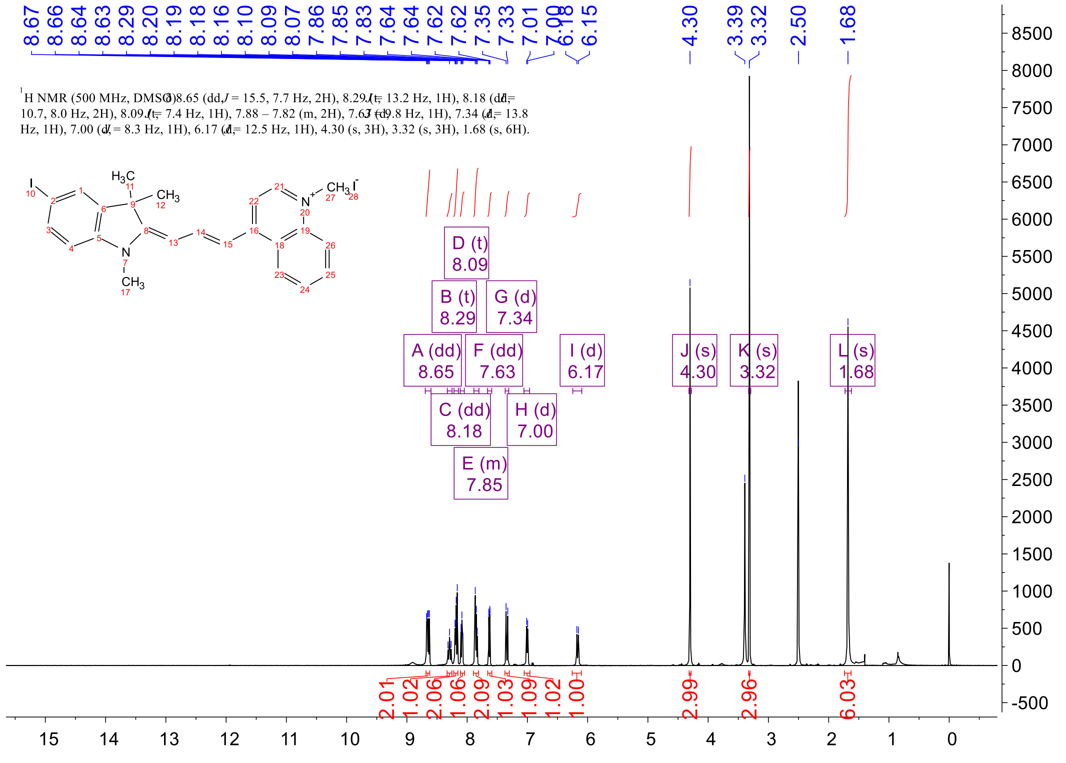

Figure S5. ¹H NMR spectrum of **QICY** recorded in DMSO-*d*₆.

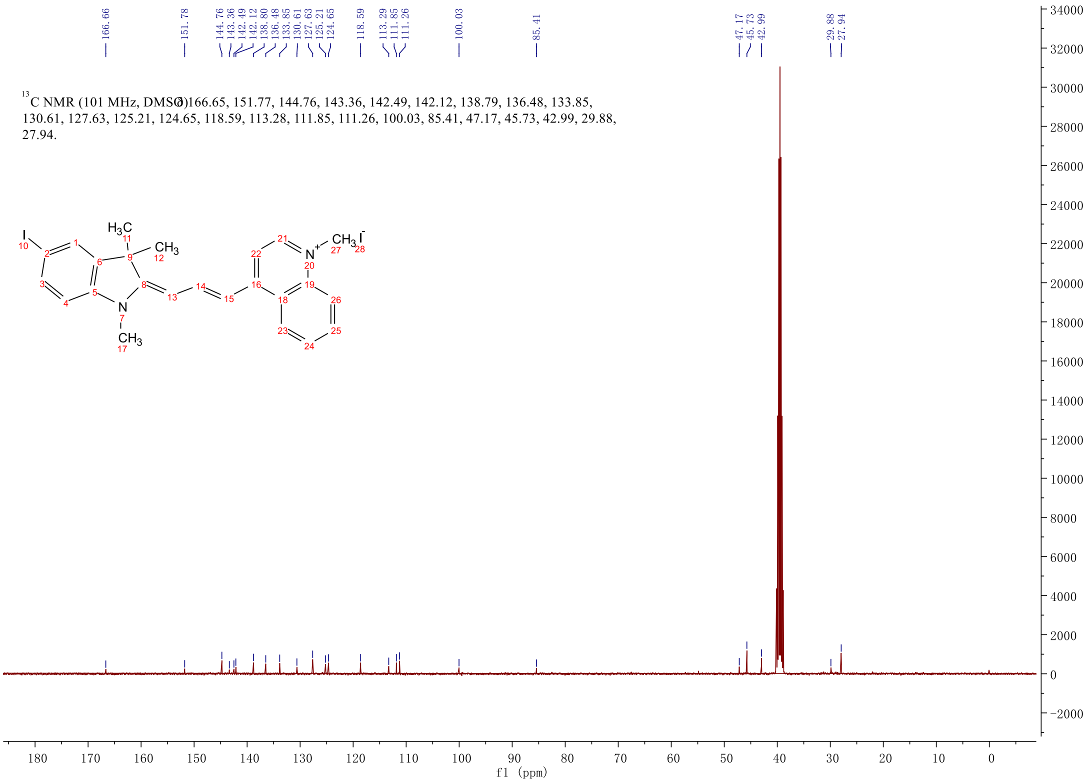

Figure S6. ^{13}C NMR spectrum of **QICY** recorded in DMSO-d_6.

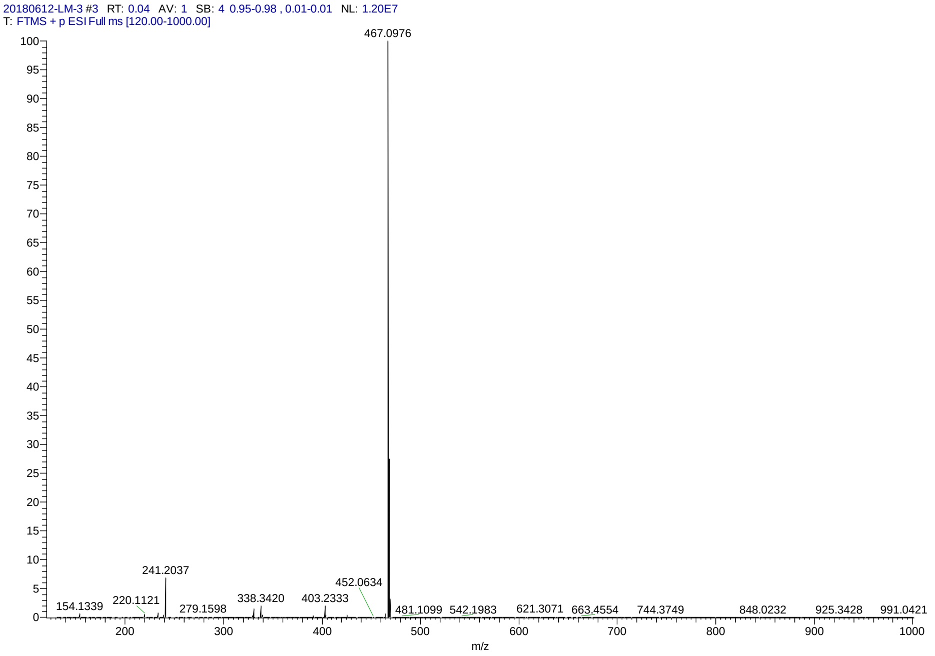

Figure S7. ESI mass spectrum of compound **QICY**.

3. Subcellular Localization Assay

The investigation of QICY subcellular localization were firstly explored by the co-localization assays with commercial organelle dyes Mito-tracker® Green FM and Hoechst 33342 for organelle localization, and then with SYTO® RNASelectTM green fluorescent cell stain and Hoechst 33342 for component localization. SYTO® RNASelectTM green fluorescent cell stain and Hoechst 33342 are selective for intracellular RNA and dsDNA separately. QICY and the commercial organelle trackers were successively added to the same dish and cultured with cells at 37°C for different duration in culture medium. The culture concentrations and working procedures of commercial trackers can be acquired from production instruction books provided by reagent companies. After incubation, cells were washed with PBS three times after discarding the culture medium, and 1 mL serum-free medium was finally added before imaging. Cells were imaged with a 100× oil-immersion objective lens in an inverted-type scanning microscope (Olympus FV3000). To avoid the cross-color interference, the fluorescence of QICY, RNA stain and Hoechst 3342 were excited and collected in the different phases of confocal laser scanning microscope. The laser wavelengths to excite QICY, commercial RNA stain and Hoechst 3342 and the corresponding collection ranges of fluorescence emission are listed in the Table S1.

Table S1. Imaging conditions of QICY, RNA stain, and Hoechst 3342 in CLSM

	λex (nm)	λem (nm)
QICY	640	650- 750
Mito-tracker® Green FM	488	510-540
SYTO®RNASelect™	488	520- 600
Hoechst3342	405	430- 470

4. Experimental results

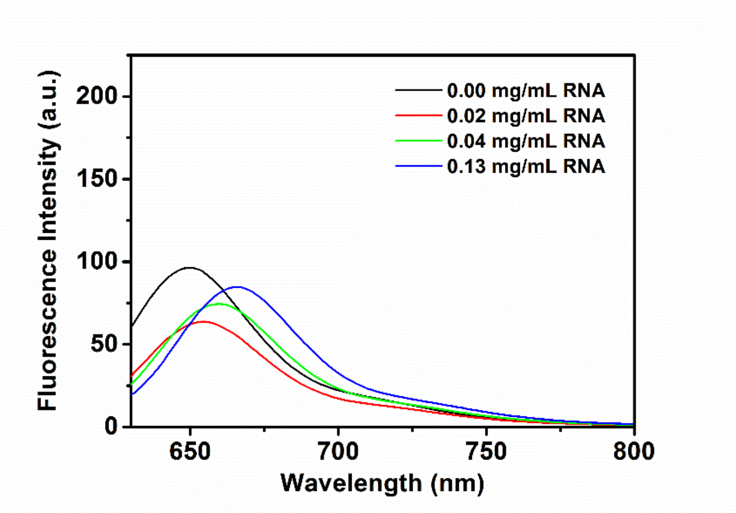

Figure S8. Influence of RNA titration on the fluorescence emission spectra of **QICY** (1.3 μM) in ultrapure water.

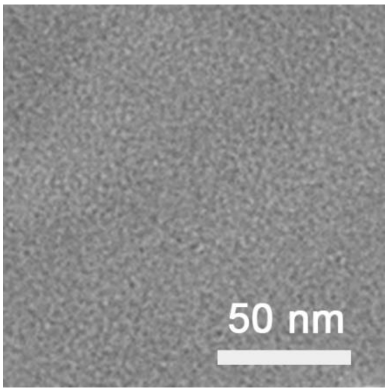

Figure S9. No **QICY** aggregates observed by TEM before interaction with RNA in ultrapure water.

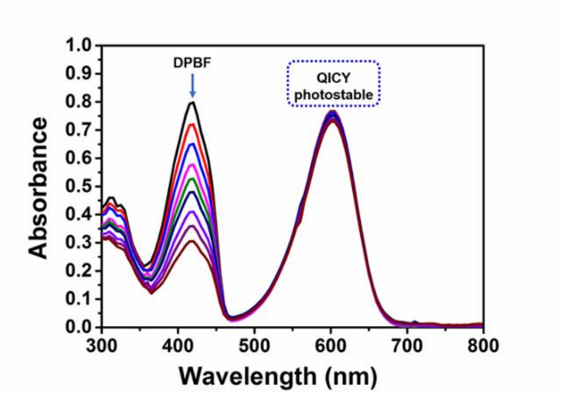

Figure S10. Absorbance change of **QICY** and DPBF in DMSO with irradiation time.

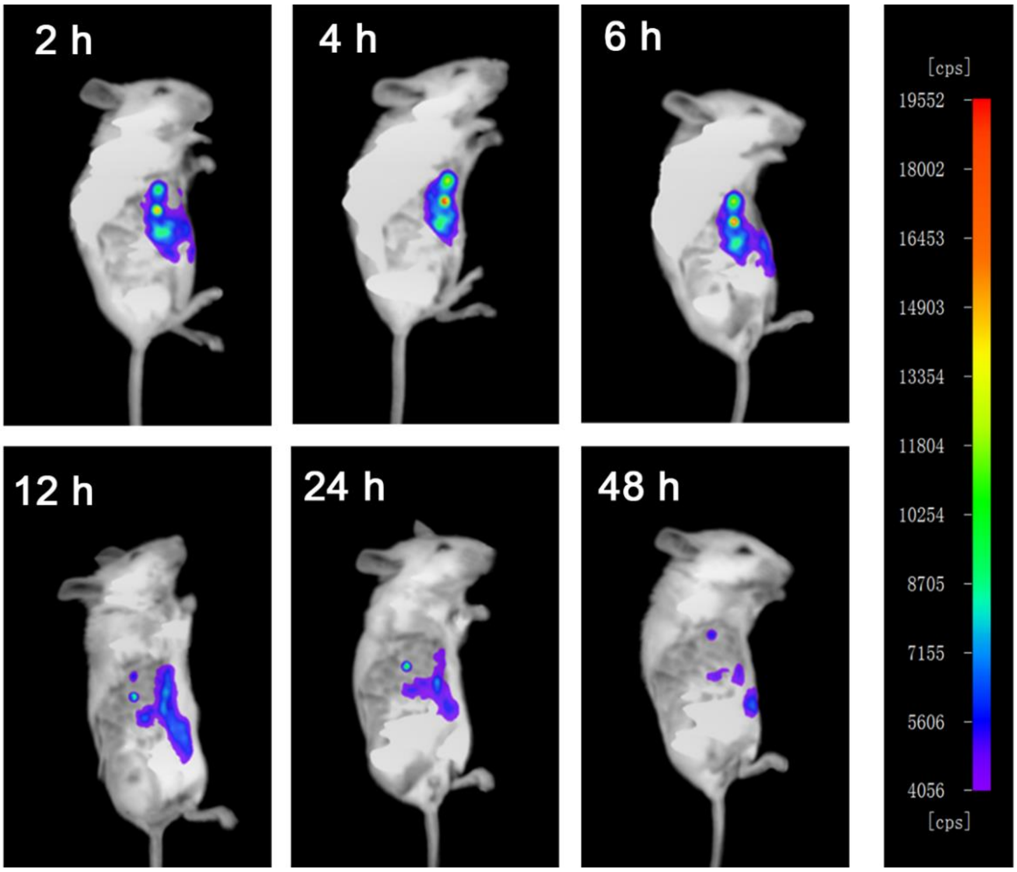

Figure S11. *In vivo* fluorescence imaging of 4T1 tumor-bearing BALB/c mice after the intravenous injection of **QICY** at the concentration of 400 μM. Ex. = 630 nm/ Em. = 700 nm.

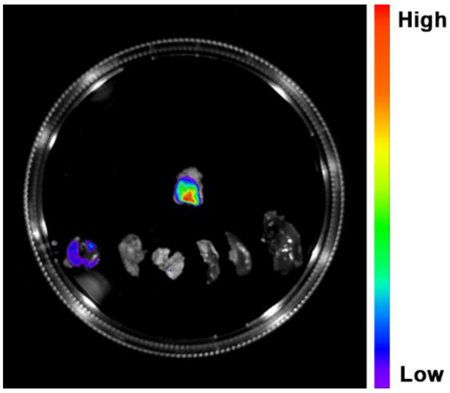

Figure S12. Fluorescence imaging of various organs (from top to bottom, left to right: tumor, kidney, colon, lung, heart, spleen, and liver) at 4 h after the intravenous injection of **QICY** (tail vein injection, 400 μM, 100 μL, 1.73 mg/kg).

References

(1) Atchison, J.; Kamila, S.; Nesbitt, H.; Logan, K. A.; Nicholas, D. M.; Fowley, C.; Davis, J.; Callan, B.; McHale, A. P.; Callan, J. F. Iodinated cyanine dyes: a new class of sensitisers for use in NIR activated photodynamic therapy (PDT). *Chem. Commun.* **2017**, *53*, 2009-2012.
(2) Klotz, E. J. F.; Claridge, T. D. W.; Anderson, H. L. Homo- and hetero- 3 rotaxanes with two pi-systems clasped in a single macrocycle. *J. Am. Chem. Soc.* **2006**, *128*, 15374-15375.
(3) Peng, X. J.; Wu, T.; Fan, J. L.; Wang, J. Y.; Zhang, S.; Song, F. L.; Sun, S. G. An Effective Minor Groove Binder as a Red Fluorescent Marker for Live-Cell DNA Imaging and Quantification. *Angewandte Chemie-International Edition* **2011**, *50*, 4180-4183.
